# Foreign aid or foreign investments: call for a paradigm shift in mentality and nomenclature

**DOI:** 10.11604/pamj.2015.20.214.6174

**Published:** 2015-03-10

**Authors:** Obinna Ositadimma Oleribe, Okey Nwanwanyu

**Affiliations:** 1 Excellence & Friends Management Care Center (EFMC), Abuja, Nigeria; 2 Global Health Services Network LLC, Michigan

**Keywords:** Foreign aid, foreign investments, donor

## Abstract

Funding for health care programs has over the years been an important challenge for health and health care services. However with the advent of financing, part of this problem was resolved. Through these investments, lives were saved, many destinies recovered and some obsolete systems reengineered. Major proofs of these expenditures are number of people reached and sometimes number of sites opened/supported, which in several cases, are not entirely verifiable. Sustainable development from these funds is limited, and far and in between. This is despite the fact that supports for health care and health care services have been ongoing for more than 60 years. As long as these funds are seen as aids to developing countries, they will continue to fail to achieve their primary objectives. But looking at these as investments in supported countries will significantly improve the outcome, health system impacts, as well as engineer sustainable health system strengthening and improvement. Such a re-branding will reduce the politics of support, improve effectiveness and efficiency in the use of the resources, and empower receiving nations towards better health systems.

## Commentary

Several years ago, the greatest problem in health care was the scarcity of funds to implement health programs, prevent diseases and provide care and support services. But, today, all this has changed thanks to the various international funders whose remarkable generosity has led to an unprecedented rise in resources made available for various health programs across the world. However, these funds have failed to achieve the primary goals for which they were established both in Africa and the rest of the world due to several administrative and implementation errors [1]. One of the major errors of implementation is that these funds are seen as foreign aid to receiving nations and not as investments by the granting nations. In this simple reflection, we want to consider this further. Over the years, developed countries have supported several developmental activities in low and middle income countries across the world. This has gone on for more than 60 years since the first Official Development Assistance (ODA) program was instituted [2]. Nigeria and nations in the sub-Saharan region have benefited massively from these funds. In HIV programs, funds were received through the US President's Emergency Plan for AIDS Relief (PEPFAR), Global Fund (GF) and several bilateral and multilateral agreements to provide testing services, anti-retroviral therapy and other supportive care. Over the past two decades, billions of US dollars have been spent in health, environmental issues, democracy and governance, justice and human rights, and several other projects. With these supports came also the need to meet the requirements of the funders, both administrative and technical. Most supported programs in health were aimed at disease eradication (poliomyelitis), elimination (malaria) or control (HIV). From these investments, lives were saved, many destinies recovered and some obsolete systems reengineered.

The recent Ebola viral disease (EVD) outbreak in West Africa, however, called into question the real and sustainable benefits of these investments or aids. With billions of US dollars expended in health, the public health systems in most sub-Saharan African nations are still very rudimentary. Furthermore, these are the major beneficiaries of these investments. Human resource for health in these nations remains among the worst worldwide as capacity of the health system remains inadequate to meet public health emergencies of global importance, and health outcomes are still very unacceptably poor. These outcomes are not unexpected as these funds are programmed to respond to specific health conditions rather than the public health system in general. Also, as the goals of these funded public health projects are driven by external agencies with specific agendas and categorical funding, community organizing, involvement and ownership is impossible. These funding mechanisms were initiated to reduce inequity in the system, minimize deaths from preventable diseases and achieve short-term public health goals. Effective use of these funds has helped build organizations like Excellence and Friends Management Care Centre (EFMC) and several others, providing jobs for several jobless youths, providing services to majority who may never have had access to care and support, building the capacity of health care workers, and even renovating health care facilities in supported states of Nigeria. Are these merely financial aids from developed countries or financial investments? As the world becomes a single global village, a person can have breakfast in Abuja, lunch in London and dinner in New York. This means that what happens in one part of the world directly and indirectly affects the rest of the world. The EVD is a good case in point. In December 2013, it was simply a Guinean problem. But by January 2015, it had affected nine different nations including the United States and United Kingdom, with close to 21,000 documented cases and 8,235 deaths [3]. Similarly, peace in Nigeria, for instance, will promote development in Nigeria, Africa and in most other parts of the world. Until the last case of poliomyelitis is identified and the virus eradicated, no part of the world is free of a potential outbreak. In the same vein, until we contain HIV and ensure that there are no new infections anywhere in the world, no single individual is truly protected from the infection. Thus, the need for all nations of the world to join hands, invest for healthier nations and people, so as not to allow the infectious agents launch an assault on their people. The politics of aid and donor support is also very interesting. While the funds are given, for instance, for public health work in the recipient country, the majority may be spent in the donor country building systems and structures; and data are collected for scientific research. A number of seminar research publications are based on data obtained from funded projects in the developing world. These funds, therefore, provide opportunity to build the capacity and enhance the experience of funders’ citizens - from interns, research fellows to professors on tropical diseases, developing systems and processes; which serve as platforms for multiple scientific articles, publications and conference presentations. In addition, individuals in the developing countries are used as specimens/cases in pilot tests, field trials, and all other experiments - even in the early phases of the trials. Moreover, most materials and equipments used in these funded projects are sourced and procured from donor nations, catalyzing industrial development. The current China railway project in Nigeria is a good case in point. China gave funds to Nigeria (loan or grant); and these funds have also provided employment for their citizens who are now working in Nigeria as expatriates, building railway stations and coaches for Nigeria while several Nigerian professionals remain jobless. In addition, all materials and equipments needed for these projects are sourced from China and imported into Nigeria with hard earned foreign currency. One day, Nigeria may be called upon to pay back.

Do we, therefore, have developed countries giving aid to developing countries? Or are these investments in developing countries? I tend to believe that they are investments more than just aid. If therefore we understand them as investments, it behoves us all to ensure that the right processes are put in place and followed, that the right outcomes are demanded and documented, and that the right strategies are used - both by the givers and the receivers. We, therefore, want to propose a paradigm shift both in terminology and practice. While the investing nations (formally called donor nations) should ensure effective and efficient use of all invested resources, with appropriate documentation of expected and actual outputs, outcomes, and impacts; focus nations (formally called benefiting nations) should ensure technical and allocative efficiency and proper management of the invested funds. Unless this is done, provided funds will only serve the interest of a few while these nations, like Nigeria, remain where she is, or even deteriorate health wise. Such paradigm shift will allow for better coordination of the funds, their use and application. Also, the focus nations’ involvement in the identification of problems/needs and the management of the funds will enhance allocative and technical efficiency, improve outcomes and enhance the public health system. Maybe one day, the capacity of the health system will be sufficiently developed to meet the needs of the people. The question is how do we use these funds to develop sustainable public health systems in Nigeria? Dambisa Moyo, in her classic book, Dead Aid, asserted that for 50 years, more than US $1 trillion in development-related aid has been transferred from rich countries to Africa without commensurate improvement in the lives of Africans [4]. According to her, it made their situation worse in some cases. Looking at these funds as investments will lead to better outcomes for both the investors and the focus nations. Failure of these projects may also be linked to the attempts by funders to impose uniform, universal, and rigid administrative systems and procedures on project organizations in developing countries [5]. In line with Rondinelli's suggestion, funders should examine the culture, needs and conditions of focus communities and tailor administrative and organizational solutions to them with their participation and collaboration. Furthermore, as new aid management strategies, while necessary, will not be sufficient to remedy the fragmentation of the health sector, a new model of collaboration between expatriate aid workers and local counterparts in the developing world is urgently needed [6]. This new model must center on building long-term equitable relationships in a sustainably funded public health sector [6]. Thus, there is need to see these not as aid, but as investments with dual responsibilities for implementation, management and outcome evaluation.
